# A Case of Chondrosarcoma That Primarily Developed in the Cervical Spine

**DOI:** 10.5812/iranjradiol.6344

**Published:** 2012-03-25

**Authors:** Ali Hekmatnia, Amirhossein Ghazavi, Bahram Aminmansour, Parvin Mahzouni

**Affiliations:** 1 Department of Radiology, Image Processing and Signal Research Center, Alzahra Hospital, Isfahan University of Medical Sciences, Isfahan, Iran; 2 Department of Radiology, Isfahan University of Medical Sciences, Isfahan, Iran; 3 Department of Neurosurgery, Alzahra Hospital, Isfahan University of Medical Sciences, Isfahan, Iran; 4 Department of Pathology, Alzahra Hospital, Isfahan University of Medical Sciences, Isfahan, Iran

**Keywords:** Chondrosarcoma, Cervical Vertebrae

Dear Editor,

Hereby, we will report a rare case of cervical spine chondrosarcoma producing a dumbbell shape foraminal mass lesion. Generally, spinal neural foraminal widening has several causes. They may be tumoral such as neurogenic tumors, developmental or vascular. Occasionally spinal neural foraminal widening is in the shape of a dumbbell lesion. In this case, the differential diagnosis is limited but a little more different. Neurogenic tumors (schwannoma and neurofibroma) are the most common.

At times some other slow-growing tumors may cause dumbbell-shaped neural foraminal widening. Other slow-growing tumors such as chordomas, ependymomas and meningiomas may every so often cause neural foraminal widening [1].

A case report of dumbbell-shaped lymphangioma, a case report of dumbbell-shaped chordoma and another case of lipoma have also been documented [2][3][4].

We report a patient with a spinal chondrosarcoma passing through the C4-C5 foramen, a dumbbell chondrosarcoma representing a rare condition.

This rare case has significant importance because it makes the radiologist think of a wider differential diagnoses of a dumbbell-shaped spinal lesion including those from the bone origin than simply neurogenic tumors and to get familiar with imaging aspects of such a condition.

Primary chondrosarcoma occurs mainly between the ages of 30 and 70 years. Although our patient is in this range, he seems to be a little young for chondrosarcoma. In the literature review, there is only one reported case of dumbbell-shaped chondrosarcoma in the cervical spine. However, the article has less focus on the imaging aspects of the tumor [2].

A 36-year-old man presented with progressive pain of left-sided upper limb for over 3 years. The pain began 3 years ago and had aggravated during the last 6 months. In his physical examination, the pain did not have a radicular characteristic. Reflexes and muscle forces were in the normal limit. The pain slightly got worse at night.

Magnetic resonance imaging (MRI) of the cervical spinal column with 1.5 Tesla magnet on T1-weighted (TR = 700.0 and TE = 12.0) and T2-weighted (TR = 3520.0 and TE = 95) sequences without contrast material reveals a lobulated dumbbell-shaped soft tissue mass with a regular border originated from the neural arcus of the fifth cervical vertebra extending to the right-sided C4-C5 neural foramina. It has low to intermediate signal intensity on T1-weighted axial (Figure 1A) and coronal (Figure 1B) images and high signal intensity on T2-weighted axial (Figure 1C) and T2- weighted coronal images (Figure 1D and E).

At surgery, the anterior part of the neck was approached through the sternocleidomastoid muscle. Deep to the muscle, there was a firm and hemorrhagic carpet-like expanded mass. It was too difficult to resect the whole tumor; so it was partially resected. Inside the mass was softer and more resectable. The family history and a skeletal survey of the patient did not reveal any other lesion.

**Figure 1:**
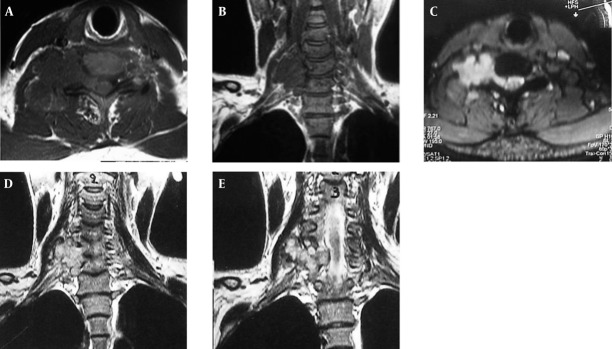
Magnetic resonance imaging of the cervical spinal column with 1.5 Tesla magnet on T1-weighted and T2-weighted sequences with contrast material reveals a lobulated dumbbell-shaped soft tissue mass with regular border originated from the neural arcus of the fifth cervical vertebra extending to the right-sided C4-C5 neural foramina. It has low to intermediate signal intensity on T1-weighted axial (A) and coronal (B) images and high signal intensity on T2-weighted axial image (C) and T2-weighted coronal image (D & E).

The pathologic study of the specimen with H & E staining and on high power view showed thin anastomosing strands of tumor cells surrounded by an abundant myxoid matrix indicating a chondrosarcoma (Figure 2).

**Figure 2:**
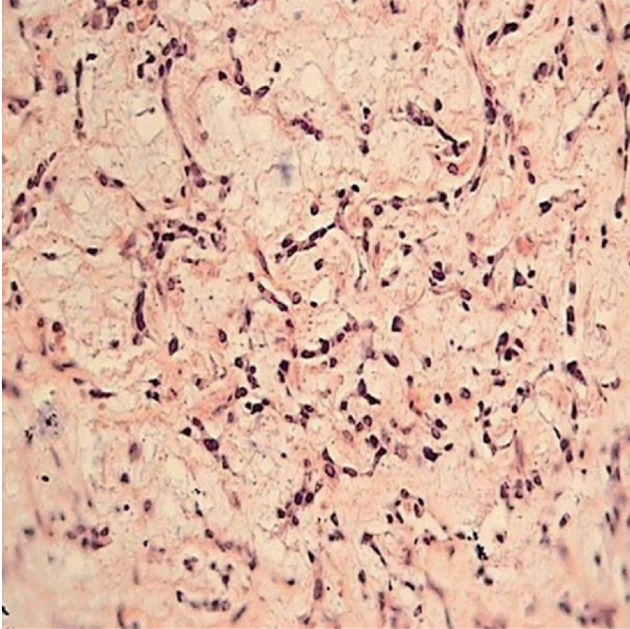
Thin anastomosing strands of tumor cells surrounded by an abundant myxoid matrix (high power view) on H & E staining indicating a chondrosarcoma.

Dumbbell tumor of the spine is not uncommon. Spinal neural foraminal widening may be due to neoplasm, arterial abnormalities or developmental causes [5][6][7]. The most common cause is the so-called dumbbell schwannoma or neurofibroma.

In the spine, a lesion with combined intradural and extradural components may lead to the so-called dumbbell mass, which passes through the corresponding neural foramen. The most common causes of spinal dumbbell lesions are schwannomas and neurofibromas, while other nerve sheath tumors, ganglioneuromas and neurofibrosarcomas are rare. Conjoined nerve roots, root sleeve cysts, ganglion cysts and foraminal disc herniations are relatively common conditions which can resemble nerve sheath tumors.

Rare causes include aneurysm, looping or tortuosity of the vertebral artery, a developmental condition associated with absence of vertebral pedicle and resultant widening and craniocaudal elongation of the neural foramen and a dumbbell-shaped lipoma.

The MRI characteristics such as signal intensity on various sequences and the pattern of enhancement in association with large paraspinal soft tissue mass suggest a sarcomatous change.

The most common site of chondrosarcoma is the pelvis and ribs. However, spinal column is an occasional site [7].

The prognosis of chondrosarcoma is relatively good if complete surgical excision is possible before dissemination. Metastasis only occurs in later stages by the hematogeneous route. In our patient, no metastatic site was documented [7].

## References

[1] Saito T, Terada K, Tsuchiya K, Oda Y, Tsuneyoshi M, Iwamoto Y (1999). Lymphangioma presenting as a dumbbell tumor in the epidural space of the lumbar spine. Spine (Phila Pa 1976)..

[2] Sakayama K, Kawatani Y, Kidani T, Sugawara Y, Miyazaki T, Fujibuchi T (2004). Dumbbell-shaped chondrosarcoma that primarily developed in the cervical spine: a case report. J Orthop Sci.

[3] Alam AM, Shuaib IL, Ghimire R (2005). Cervical dumbbell meningioma and bilateral acoustic schwannoma in a patient with neurofibromatosis type 2. Eur J Radiol.

[4] Karakida O, Aoki J, Seo GS, Ishii K, Sone S, Nakakouji T (1996). Epidural dumbbell-shaped chordoma mimicking a neurinoma. Pediatr Radiol.

[5] Osborn AG (1994). Diagnostic neuroradiology.

[6] Resnick D, Niwayama G (1981). Diagnosis of bone and joint disorders: with emphasis on articular abnormalities.

[7] Sutton D (2003). Textbook of Radiology and Imaging.

